# A geographic identifier assignment algorithm with Bayesian variable selection to identify neighborhood factors associated with emergency department visit disparities for asthma

**DOI:** 10.1186/s12942-020-00203-7

**Published:** 2020-03-18

**Authors:** Matthew Bozigar, Andrew Lawson, John Pearce, Kathryn King, Erik Svendsen

**Affiliations:** 1 Division of Epidemiology, Department of Public Health Sciences, Medical University of South Carolina, Charleston, SC USA; 2 Division of Biostatistics, Department of Public Health Sciences, Medical University of South Carolina, Charleston, SC USA; 3 Division of Environmental Health, Department of Public Health Sciences, Medical University of South Carolina, Charleston, SC USA; 4 Department of Pediatrics, Medical University of South Carolina, Charleston, SC USA; 5 School-Based Health, Center for Telehealth, Medical University of South Carolina, Charleston, SC USA

**Keywords:** Bayesian spatio-temporal modeling, Geographic imputation, Respiratory diseases, Social determinants of health, Air pollution, Hospitalization record data, Rural health, Urban health, SEA-AIR Study

## Abstract

**Background:**

Ecologic health studies often rely on outcomes from health service utilization data that are limited by relatively coarse spatial resolutions and missing geographic information, particularly neighborhood level identifiers. When fine-scale geographic data are missing, the ramifications and strategies for addressing them are not well researched or developed. This study illustrates a novel spatio-temporal framework that combines a geographic identifier assignment (i.e., geographic imputation) algorithm with predictive Bayesian variable selection to identify neighborhood factors associated with disparities in emergency department (ED) visits for asthma.

**Methods:**

ED visit records with missing fine-scale spatial identifiers (~ 20%) were geocoded using information from known, coarser, misaligned spatial units using an innovative geographic identifier assignment algorithm. We then employed systematic variable selection in a spatio-temporal Bayesian hierarchical model (BHM) predictive framework within the NIMBLE package in R. Our novel methodology is illustrated in an ecologic case study aimed at identifying neighborhood-level predictors of asthma ED visits in South Carolina, United States, from 1999 to 2015. The health outcome was annual ED visit counts in small areas (i.e., census tracts) with primary diagnoses of asthma (ICD9 codes 493.XX) among children ages 5 to 19 years.

**Results:**

We maintained 96% of ED visit records for this analysis. When the algorithm used areal proportions as probabilities for assignment, which addressed differential missingness of census tract identifiers in rural areas, variable selection consistently identified significant neighborhood-level predictors of asthma ED visit risk including pharmacy proximity, average household size, and carbon monoxide interactions. Contrasted with common solutions of removing geographically incomplete records or scaling up analyses, our methodology identified critical differences in parameters estimated, predictors selected, and inferences. We posit that the differences were attributable to improved data resolution, resulting in greater power and less bias. Importantly, without this methodology, we would have inaccurately identified predictors of risk for asthma ED visits, particularly in rural areas.

**Conclusions:**

Our approach innovatively addressed several issues in ecologic health studies, including missing small-area geographic information, multiple correlated neighborhood covariates, and multiscale unmeasured confounding factors. Our methodology could be widely applied to other small-area studies, useful to a range of researchers throughout the world.

## Background

### Geographic data in ecologic health studies

Understanding the influence of contextual factors on health, such as surrounding neighborhood characteristics, is an ongoing challenge further complicated by leveraging and integrating multiscale geographic information [1–4]. Geographic health effects studies have been conducted at multiple spatial scales using a variety of spatial units in analyses. But, commonly used administrative units (e.g., states, counties in the United States, US) are coarse, and mail-delivery units (e.g., ZIP codes) are irregular, often delimit heterogeneous population groups, and change frequently over time [5, 6]. Conversely, neighborhoods tend to delineate demographically homogenous subpopulations [7], improving the differentiation of population level risk factors. Yet, neighborhoods are challenging to incorporate in health studies.

Neighborhood units, or proxies for them (e.g., US census tracts), are often spatially misaligned with common administrative units. In addition, accurately geocoding an individual’s address to the proper neighborhood unit for use in large scale health studies presents challenges. For example, many people have non-standard address structures, particularly those living in rural areas [8, 9]. Often, researchers will either remove records from analyses that could not be geocoded to a small area, or “scale-up” entire analyses to larger spatial units at a coarser spatial scale. These decisions can introduce bias (e.g., geographic selection bias, and “cartographic confounding”) and reduce precision of the estimated associations [9]. When health studies use areal units in analyses, they must also contend with the modifiable areal unit problem (MAUP), in which the delineation of boundaries affects the values (e.g., percent of the population living in poverty) within the unit [10, 11]. There are additional limitations in areal analyses, including coarse spatial resolution, assumptions about distributions within spatial units, temporal boundary changes, and boundaries that are arbitrary for health research [12]. As such, health researchers often prefer point process or grid-based analyses that can overcome areal unit limitations. However, researchers are often limited by data format availability, making transitions to continuous or grid-based analyses inefficient or infeasible. In such cases when ideal data are not available, there is a need to develop methodologies that improve the utility of common, existing health service utilization data sources.

### Geographic missingness

Geographic missingness, or missing geographic identifiers, is different than other types of missing information, such as missing covariates. While many researchers have addressed covariate missingness easily and efficiently in Bayesian hierarchical models (BHM) [13–16], the problem of having a lack of information to properly assign a person’s residence to a discrete spatial unit has only been addressed by a few researchers [17–19]. Researchers have previously assigned geographic identifiers based on proportions of population centroids within larger spatial units [17], regressed areal sociodemographic measures on proportions of cases with missing geographic identifiers [9], and various other deterministic and stochastic allocation methods [19]. Few health researchers, however, have assessed how geographic missingness affects the identification accuracy of neighborhood risk factors that are predictive of adverse health outcomes.

### Spatio-temporal Bayesian frameworks

Continued computational improvements have allowed researchers to build increasingly sophisticated spatial or spatio-temporal models [20–23]. Recent studies have employed Bayesian small-area statistical methods that can improve flexibility of models to incorporate structured and unstructured random effects in a hierarchical framework, such as a BHM [24–26]. Controlling for unmeasured confounding by assigning variation in the outcome to either spatial, temporal, and/or spatio-temporal effects, rather than to an error term, has two important benefits: (1) the overall model fit improves, and (2) covariate coefficient estimates in the model are more precise [27–30]. Yet, few health researchers have leveraged a Bayesian predictive framework to conduct a systematic variable selection procedure for correlated neighborhood covariates. Fortunately, Bayesian variable selection approaches automatically adjust for multiplicity, or multiple comparisons [31]. Furthermore, scholars have recently called for only strategic use of health indices (e.g., neighborhood deprivation indices), instead lobbying for measuring specific neighborhood components with potential for public health intervention [32].

### Research gaps addressed

In this context, “neighborhoods” are understood as the physical and environmental conditions in which a person lives, and the ways in which neighborhoods effect health outcomes are complex [3, 33, 34]. Common limitations of using administrative health data are missing geographic information and identifying important neighborhood predictors of adverse health outcomes. Addressing such limitations, we developed a geographic identifier assignment algorithm and used a variable selection procedure, a novel combination that enhanced our spatio-temporal BHM predictive framework. We analyzed pediatric emergency department (ED) visits for asthma in the US state of South Carolina as a case study. Our primary methodologic goal was to evaluate and address geographic missingness and variable selection on two datasets from our case study: (1) using only records with valid census tract identifiers, and (2) using all records by employing a geographic identifier assignment procedure. Our subsequent public health goal was to identify and detail key neighborhood socioenvironmental factors and interactions associated with our outcome, neighborhood asthma ED visit risk. We show how a methodology including a geographic identifier assignment algorithm and variable selection in a flexible BHM predictive framework can improve ecologic health studies by more accurately disentangling the complex socioenvironmental factors associated with pediatric asthma disparities using existing ED utilization data.

## Methods

### Motivating data

There are well documented disparities in asthma outcomes by race, socioeconomic (SES) status, urban/rural status, and other factors, but these patterns vary by world region [35, 36]. Past research has highlighted the disproportionate asthma burden among urban children. However, recent research has shown a similar asthma-related health burden in rural areas, especially among rural African American communities [37–44]. Risk factors for asthma ED visits include factors at an individual level, in addition to socioenvironmental factors at family, neighborhood, and even regional or administrative levels, making them difficult to disentangle. Uncontrolled or severe asthma, whether due to unique individual experiences with the disease or influenced by deprived neighborhoods and poverty [45, 46], can lead patients to seek care at EDs.

**Fig. 1:**
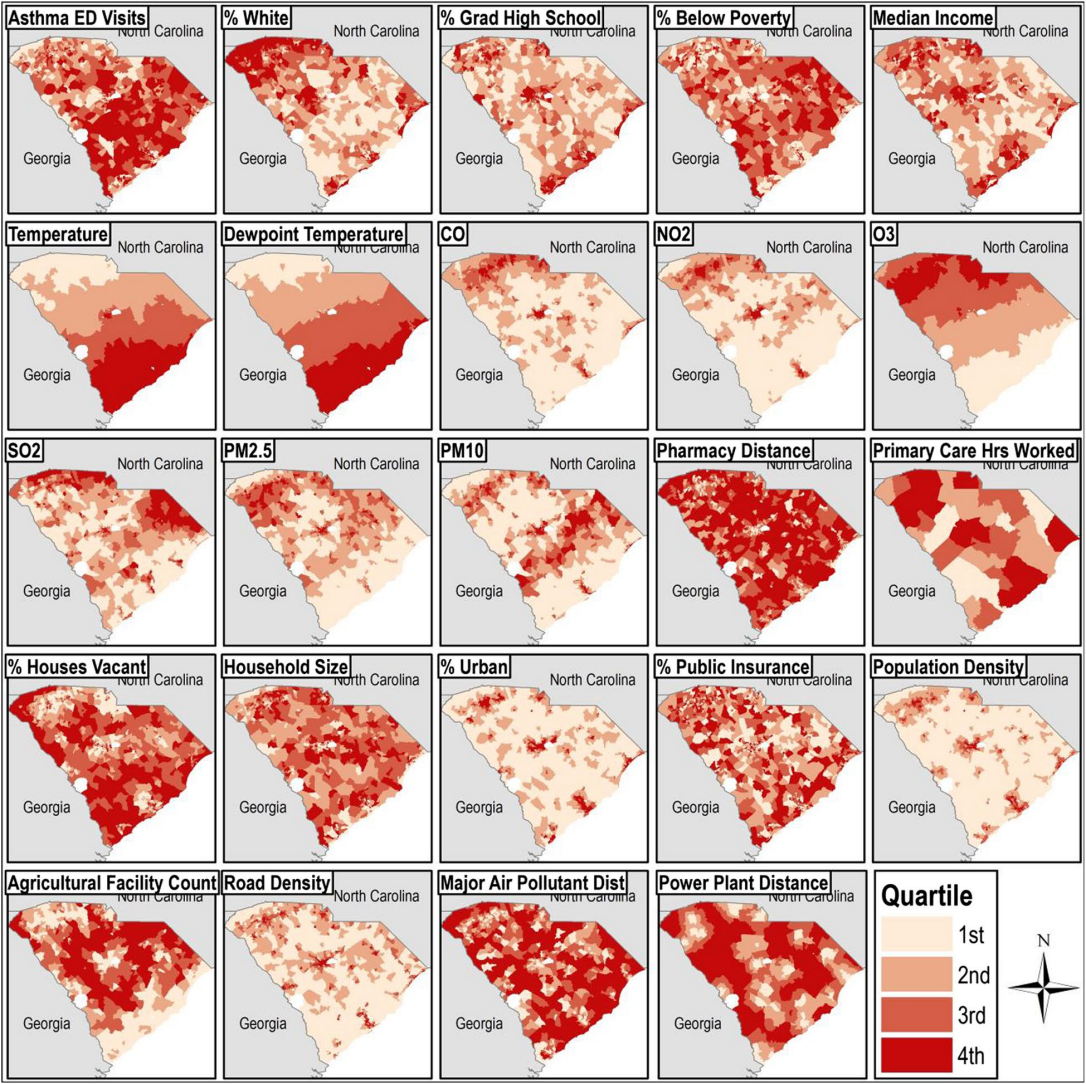
Quartiles of asthma emergency department (ED) visits and risk factors included in the study by census tract in South Carolina 1999-2015. Percent male, percent youth, direction to nearest major air pollutant facility, and direction to nearest fossil fuel burning power plant are not mapped. Annual-varying measures were averaged over time

We used data from the South Carolina Revenue and Fiscal Affairs (SCRFA) office, which combined asthma (International Classification of Disease 9, ICD9, codes 493.XX, primary diagnosis) ED visit record data from multiple sources across payor types (including no insurance). Records also included billing address geographic identifiers at the census tract and ZIP code levels. To the best of our knowledge, the dataset captures all ED visits for asthma among children ages 5–19 in the state of South Carolina for the years 1999–2015. Records were aggregated by year and census tract to generate the outcome, ED visit count per year, to facilitate a small-area ecologic analysis of counts in spatial units. Data on demographics, weather, and the 19 prospective socioenvironmental covariates and confounders (air pollutants, social, and environmental confounder categories) were attained from numerous sources listed in Table 1 and visualized in Fig. 1.^1^ Consistent with our covariate data, we selected a static geometry, 2010 US census tracts, for analytic simplicity, and all variables were joined and mapped to it [47]. We used a geographic information system (GIS) (ArcGIS, Environmental Systems Research Institute, Redlands, CA) when necessary to create measures from spatially-explicit information.

**Table 1:** Variables that were constructed and considered during the model building process

Variable	Description	Level	Type	Time	Source
Outcome					
Count	Asthma ED visit count	Census tract	Count	1999–2015	SCRFA
Demographics					
PERC_W	Percent of the population white race	Census tract	Continuous	2010	US Census
Per_You	Percent of the population ages 5–19	Census tract	Continuous	2010	US Census
Per_Mal	Percent of the population male	Census tract	Continuous	2010	US Census
Per_high	Percent of the population graduated high school	Census tract	Continuous	2010	US Census
POV100	Percent of the population < 100% federal poverty level (FPL)	Census tract	Continuous	2010	US Census
hmedinc	Household median income (scaled by $1 k)	Census tract	Continuous	2010	US Census
propmiss	Average annual proportion of census tract identifiers missing	Census tract	Continuous	1999–2015	SCRFA
Weather					
Temp	Average annual temperature (℃)	Census tract	Continuous	1999–2015	PRISM
Dewp	Average annual dewpoint temperature (°C)	Census tract	Continuous	1999–2015	PRISM
Air pollutants					
CO	Average annual CO concentration (ppm)	Census tract	Continuous	1999–2015	CACES
NO_2_	Average annual NO_2_ concentration (ppb)	Census tract	Continuous	1999–2015	CACES
O_3_	Average annual O_3_ concentration (ppb)	Census tract	Continuous	1999–2015	CACES
SO_2_	Average annual SO_2_ concentration (ppb)	Census tract	Continuous	1999–2015	CACES
PM_25_	Average annual PM_2.5_ concentration (μg/m^3^)	Census tract	Continuous	1999–2015	CACES
PM_10_	Average annual PM_10_ concentration (μg/m^3^)	Census tract	Continuous	1999–2015	CACES
Social					
Pharm_km	Distance to nearest pharmacy (km)	Census tract	Continuous	2017	SCBOP
tot_hour	Total hours worked in primary care by health professionals	County	Continuous	2018	SCRFA
PER_VAC	Percent houses vacant	Census tract	Continuous	2010	US Census
PPL_HOUSE	Average people per house	Census tract	Continuous	2010	US Census
Per_Urb	Percent of the population urban	Census tract	Continuous	2010	US Census
Ped_Per_Un	Percent of the pediatric population on public insurance	Census tract	Continuous	2010	US Census
Pop_km2	Population density (people/km^2^)	Census tract	Continuous	2010	US Census
Environmental confounders					
Ag_count	Agricultural facility count	Census tract	Count	2018	SCDHEC
Road_km2	Road density (km road/km^2^ census tract area)	Census tract	Continuous	2018	SCDOT
maj_km	Distance to nearest major air pollutant emitting facility (km)	Census tract	Continuous	2017	US EPA
maj_ang_rad	Direction to nearest major air pollutant emitting facility (radians)	Census tract	Continuous	2017	US EPA
pow_km	Distance to nearest fossil fuel burning power plant (km)	Census tract	Continuous	2017	US EPA
pow_ang_rad	Direction to nearest fossil fuel burning power plant (radians)	Census tract	Continuous	2017	US EPA

*CASES* Center for Air, Climate, and Energy Solutions [75], *PRISM* Climate Group [76], *ACS* 2010 US Census and 2010 American Community Survey [77], *SCRFA* South Carolina Revenue and Fiscal Affairs, *SCBOP* South Carolina Board of Pharmacy, *SCDOT* South Carolina Department of Transportation, *SCDHEC* South Carolina Department of Health and Environmental Control, *EPA* Environmental Protection Agency

### Geographic identifier assignment

**Fig. 2:**
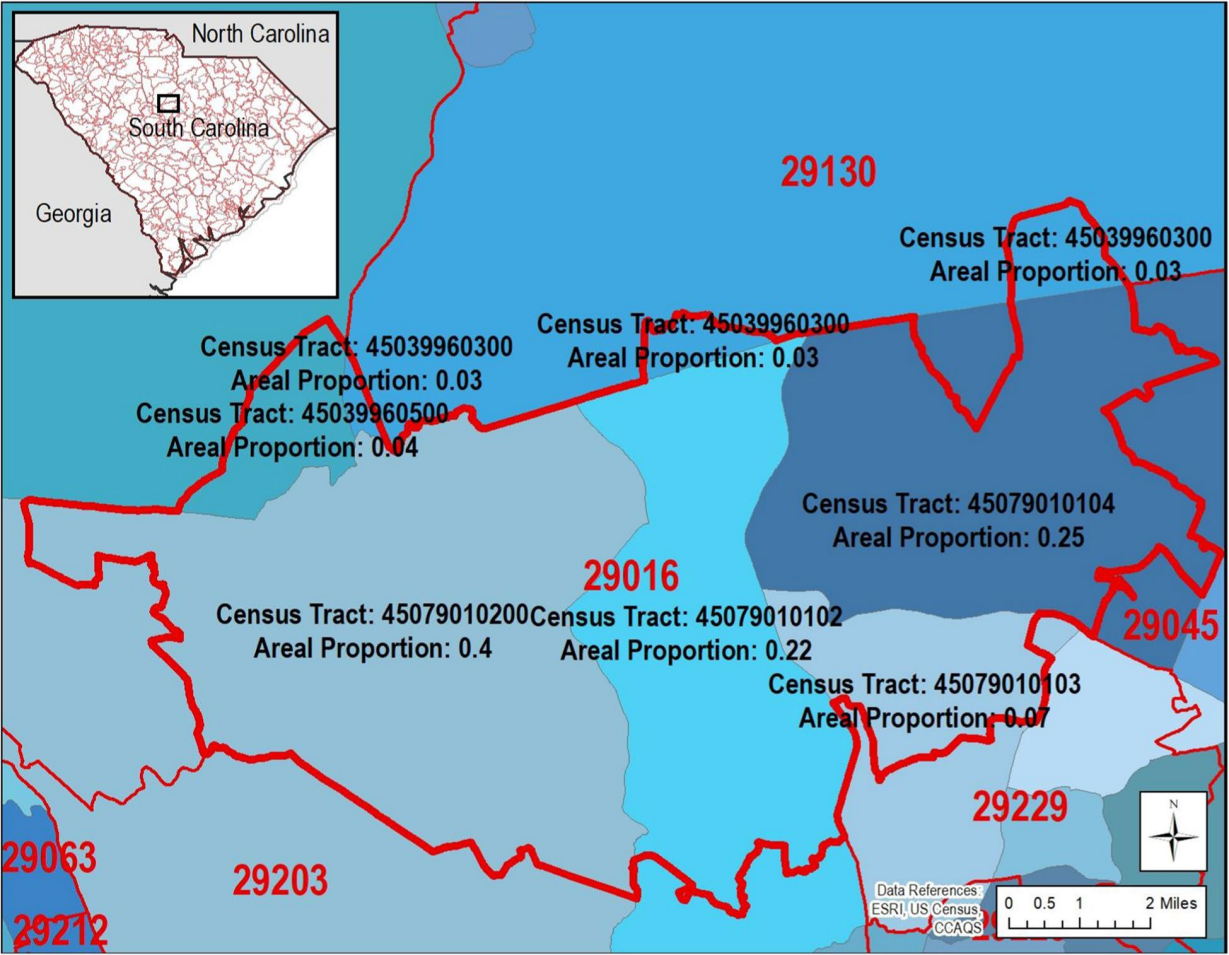
Exhibit displaying areal proportions used in the stochastic geographic identifier assignment procedure for emergency department (ED) visit records having a missing census tract identifier

We employed an intuitive, stochastic geographic imputation algorithm using areal weighting and misaligned spatial units at different scales (i.e., multiscale). Recognizing that census tract sizes are inversely proportional to population density (e.g., sparsely populated census tracts in rural areas tend to be larger in area than densely populated urban tracts), we chose to use areal proportions as probabilities of assignment to attempt to reconstruct the differential patterns of rural-dominated missingness. The algorithm consisted of 4 main steps. We (1) determined the proportion of census tract areas (generally the smaller unit at a finer spatial scale) contained within each misaligned ZIP code (generally the larger unit at a coarser spatial scale). Next, we (2) ordered the census tract-in-ZIP code proportions in a list that we set and saved, which subsequently generated a cumulative proportion. For every record with a missing census tract identifier, we then (3) generated a random number between 0 and 1, and (4) the cumulative proportion range in which it fell determined the imputed census tract identifier in its respective ZIP code (Dataset A1). An exhibit, Fig. 2, visualizes the spatial mismatch of intersected census tracts and ZIP codes.

### Statistical analyses

We constructed a spatio-temporal BHM predominately in the novel NIMBLE package in R [48–52] due to its stability and computational efficiency. This is one of the first applications of the NIMBLE package to model spatio-temporal data in a health context. We created a general hierarchical model framework that we subset to construct all other models, and it took the form:

$$Y_{ik} \sim Poisson\left( {\mu_{ik} } \right)$$

$$\mu_{ik} = E_{i} \theta_{ik}$$

1 $$\log \left( {\theta_{ik} } \right) + \alpha + u_{i} + v_{i} + w_{j} + g_{k} +\varvec{\beta}^{\prime }_{1} \varvec{X}_{ik} +\varvec{\beta}^{\prime }_{2} \varvec{X}_{j}$$

$$\beta_{*} \sim Normal\left( {0,\tau_{*}^{ - 1} } \right)$$

$$\tau_{*}^{ - 1} \sim Gamma\left( {2,1} \right)$$

in which, at the first level of the hierarchy, the Poisson-distributed observed counts, $Y_{ik}$, were modeled with rate term, $\mu_{ik}$, indexed by $i$ census tracts over $k$ years. At the second level of the hierarchy, *μ*_*ik*_ was a function of the expected counts, $E_{i}$, and relative risk, $\theta_{ik}$. The expected counts, $E_{i}$, were generated by taking the product of the observed average annual statewide asthma ED visit rate for 1999-2015 and the census tract population. Both were relatively static over time, though results were sensitive to models that used annually-varying expected counts. However, because previous research identified that a stabilized, time invariant measure of expected counts was preferable in analyses of space-time data [53], we chose the time invariant version of $E_{i}$. Using a log link shown in Eq. 1 at the third level of the hierarchy, we modeled the relative risk of ED visits in which $j$ indexed counties, $\alpha$ was the model intercept, $u_{i}$ was a correlated spatial random effect (census tract), $v_{i}$ was an uncorrelated spatial random effect (census tract), $w_{j}$ was a correlated spatial random effect (county), $g_{k}$ was a temporal trend effect (year), $\varvec{\beta}_{1}$ was a vector of coefficients (census tract), $\varvec{X}_{ik}$ was a vector of variables (census tract-years), $\varvec{\beta}_{2}$ was a vector of coefficients (county), and $\varvec{X}_{j}$ was a vector of variables (county). Few previous studies have addressed unmeasured confounding at multiple levels by including random effects at multiple spatial scales. At the fourth level of the hierarchy, $\beta_{*}$ coefficients each followed zero-centered normal distributions with precision parameters $\tau_{*}$ (variance $\tau_{*}^{ - 1}$), respectively. Finally, at the fifth level of the hierarchy, $\tau_{*}$ precisions each followed gamma distributions with shape parameter 2 and scale parameter 1, making them weakly informative [54, 55]. Furthermore, because the underlying model was a Poisson model of counts, we could not easily incorporate the individual level covariates (e.g., patient race, sex, age). The choice to use a Poisson model was made for three main reasons: we desired to (1) model a stable outcome, to (2) quantify our outcome over time and space, and (3) we lacked suitable control data (e.g., we did not have non-case information, nor did we have a suitable control disease unrelated to asthma for contrast). All variables were mean-centered to improved computational efficiency.

### Model building

We adopted the following model-building strategy: first, we assessed different versions and structures of spatial, temporal, and spatio-temporal random effects without covariates by fitting multiple preliminary models. We found that the random effects shown in Eq. 1 were the optimal combination for both model convergence and describing the variation in ED visit counts absent any covariates (Model 1a). For comparative purposes, we next examined a model with only a priori demographic and weather controls (Model 1b) and assessed if it were superior to a model with only random effects (Model 1a). We next merged Models 1a and 1b to assess whether a model with random effects, demographic, and weather covariates (Model 2) were superior to the model subsets.

### Variable selection

Few researchers have previously leveraged variable selection in analyses of health service utilization data, especially after addressing geographic missingness. A preliminary model without interactions was first fit, and Markov chain Monte Carlo (MCMC) sampling was conducted. From preliminary model results, it was determined that average annual CO concentration was a statistically significant variable. We then chose to test theoretically plausible interactions with the remaining five air pollutants (NO_2_, O_3_, SO_2_, PM_2.5_, and PM_10_) and two weather variables (temperature, dewpoint temperature) to assess effect modification, including CO as a main effect. We then fitted a Gibbs variable selection model (Model 3) employing $\phi$ entry parameters [56] following $\phi \sim Bernoulli\left( p \right)$ and having $p \sim Beta\left( {0.5,0.5} \right)$ prior distributions on the 19 prospective air pollutant, social, and environmental confounding variables (Table 1), in addition to five CO-air pollutant interactions. MCMC sampling was again conducted. Variables were selected if the respective posterior mean values of $\phi$ were greater than 0.5 (i.e., 0.5 probability, or 50% of samples) [57]. We chose to avoid a stepwise variable selection process to not introduce additional unmeasured confounding from variables that were not yet in the model. As such, we applied an all-at-once selection model to determine the collection of variables from amongst the correlated covariates that the model deemed most important in best describing variation in asthma ED visit risk.

### Model fit assessment

For each model, we quantified the log pseudo marginal likelihood (LPML), a “leave one out” cross-validated fit statistic in which less-negative values indicate better fit. Here, LPML fit statistics are calculated using the average annual conditional predictive ordinate (CPO) values for each census tract. We chose to evaluate the LPML as opposed to other statistics such as the deviance information criterion (DIC), Watanabe-Akaike information criterion (WAIC), and the mean squared prediction error (MSPE) because it was both cross-validated and computationally efficient.

### Sensitivity analyses

To assess sensitivity of subsequent analyses to the geographic identifier assignments, we repeated the algorithm four additional times, creating Datasets A2-A5. Each repetition of the algorithm allowed for each record with a missing census tract identifier the possibility to be assigned to another census tract within the record’s known ZIP code. Furthermore, we created five additional, independent Datasets A6-A10 that used a population proportion-based assignment algorithm instead of areal proportions as probabilities. For additional comparison and contrast, we also created a second dataset (Dataset B) that removed records with missing census tract identifiers—a complete case analysis. We constructed and fit models on each dataset independently. Similar to our decision on a cut point of 0.5 for $\phi$, we chose variables that were selected across Datasets A1–A5 greater than 50% (i.e., the majority) of the time. Here, unlike covariates whose values can be easily imputed multiple times (i.e., multiple imputation) quickly in BHM framework, it is highly computationally intensive to run numerous variable selection models employing sampling of the posterior distribution. Moreover, geographic identifiers are discrete and are used as the basis for determining the health outcome counts, and bear little resemblance nor function similarly to a covariate that might have missing values. Using results from the variable selection models (Model 3), we attained a final model (Model 4).

We conducted additional sensitivity analyses to assure that the results from Bayesian hierarchical space–time models were not model-driven by reproducing final Model 4 for Datasets A1-10 and Dataset B in generalized linear mixed effects (GLMM) model framework. Contrasted across frequentist and Bayesian frameworks, coefficient estimates were generally similar and in the same direction for every predictor variable.

Furthermore, we assessed sensitivity of results to specification of the CO variable. We employed a categorical quartile version of CO with the first quartile as the reference group that did not change results.

## Results

**Fig. 3:**
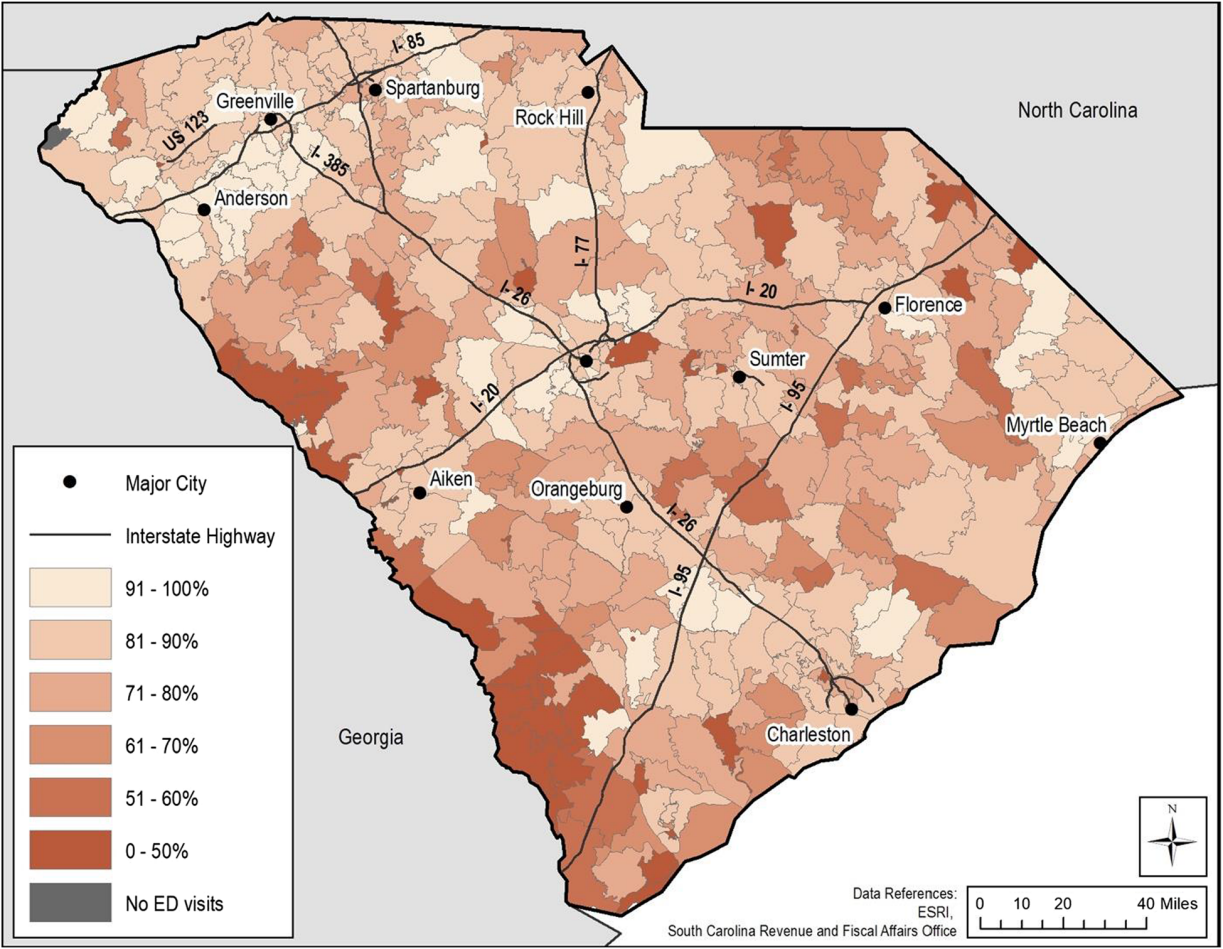
Average proportion of emergency department (ED) visits having a missing census tract identifier by ZIP code tabulation area (ZCTA) in South Carolina 1999–2015

Approximately 96% of the ED visit records had a valid ZIP code identifier, and 20% of those records had a missing census tract identifier. The primary cause of missingness in our data were mostly due to non-standard address structures more common for rural areas [8], such as PO boxes and rural routes. Significant population differences existed for individual child records having complete geographic information compared to records with missing geographic information overall (Table 2) and spatially by ZIP code tabulation area (ZCTA, Fig. 3). Records with a missing census tract identifier were significantly associated with being more African American (73.9% compared to 66.9%), more on subsidized insurance (64.1% compared to 61.5%), and visiting a rural emergency department more frequently (40.4% compared to 29.3%). Even though the children with missing census tract information more frequently visited a rural care center contrasted with children’s records having intact census tract identifiers, we posit that many others that visited urban facilities may have been rural residents. As such, rural residents still may be undercounted, indicating a large rural dimension to records with missing census tracts.

**Table 2:** Individual characteristics of emergency department (ED visits) for asthma 1999–2015 among children in South Carolina by whether records had missing or complete census tract identifiers

	Census tract identifier		
	Missing (%)	Complete (%)	p
n	21,268	96,570	
Race			
White	4725 (22.2)	27,468 (28.4)	< 0.001
African American	15,721 (73.9)	64,561 (66.9)	
Hispanic	361 (1.7)	2037 (2.1)	
American Indian	56 (0.3)	225 (0.2)	
Asian	30 (0.1)	218 (0.2)	
Other	375 (1.8)	2061 (2.1)	
Age (median [IQR])	10.00 [7.00, 14.00]	10.00 [7.00, 14.00]	0.001
Sex			
Female	9024 (42.4)	40,495 (41.9)	0.187
Male	12,244 (57.6)	56,075 (58.1)	
Payor			
Governmental	13,636 (64.1)	59,422 (61.5)	< 0.001
Private insurance	4976 (23.4)	25,577 (26.5)	
Self-pay	2551 (12.0)	11,129 (11.5)	
Other	105 (0.5)	442 (0.5)	
Urbanicity			
Urban	12,679 (59.6)	68,323 (70.7)	< 0.001
Rural	8589 (40.4)	28,247 (29.3)

**Fig. 4:**
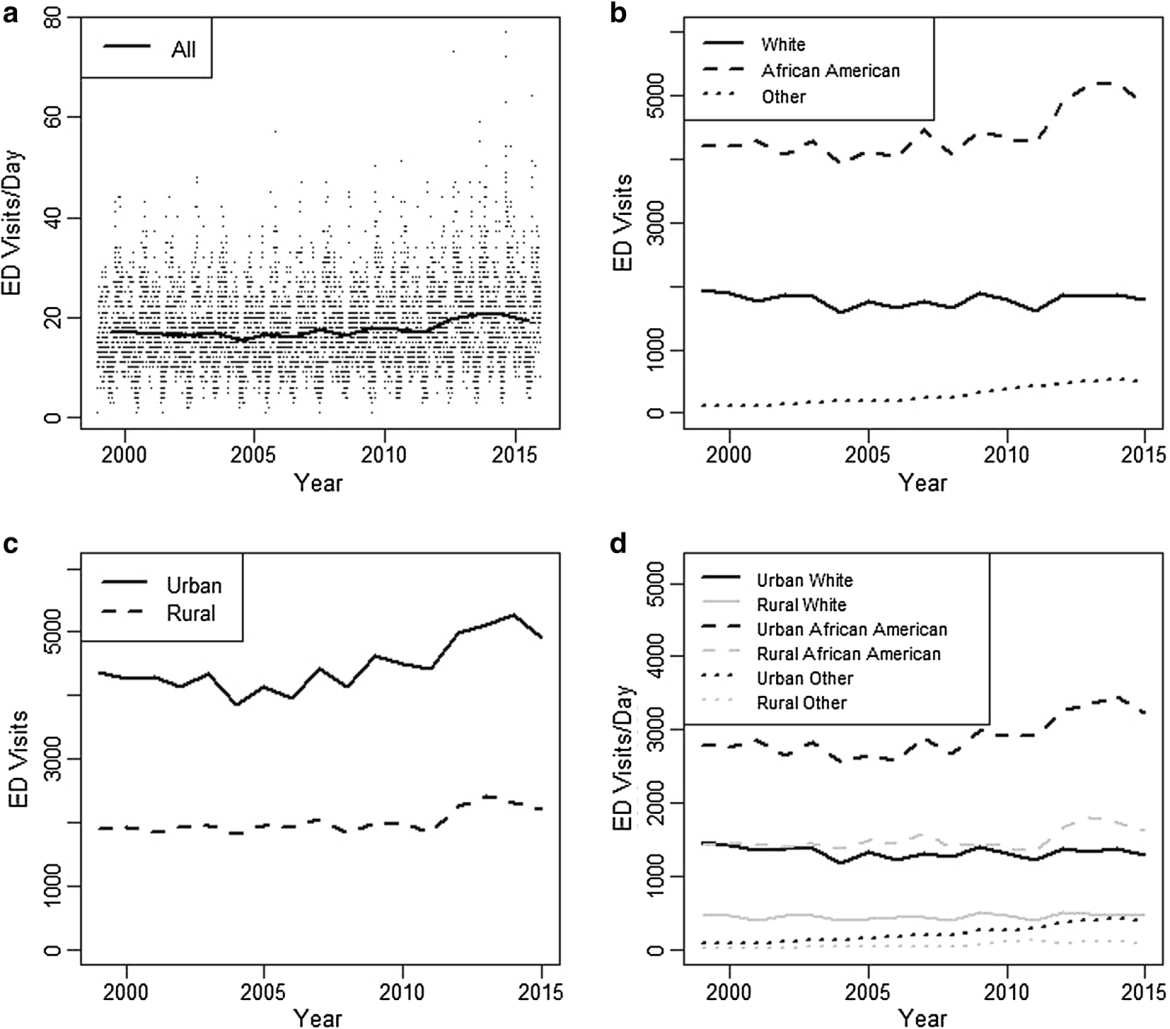
Emergency department (ED) visits in South Carolina 1999–2015. **a** All daily ED visits (points) and the annual average number of daily ED visits (line). **b** Annual sum of ED visits by patient race. **c** Annual sum of ED visits by urban/rural status of admitting ED. **d** Annual sum of ED visits by patient race and urban/rural status of admitting ED

In addition, there were patterns in the aggregated ED visit records in South Carolina for 1999–2015 (Fig. 4). A vastly disproportionate ED visit burden existed for African Americans that comprised only 27.6% of the 2010 state population (Figs. 4b and 4d) [47]. There were increases in ED visits over time, particularly among both urban and rural African Americans (Fig. 4: all panels). Note that the urban/rural status here is for the admitting facility and not patient residence. The increasing raw trend was later mirrored by the presence of positive annual temporal trend effects in statistical models.

**Fig. 5:**
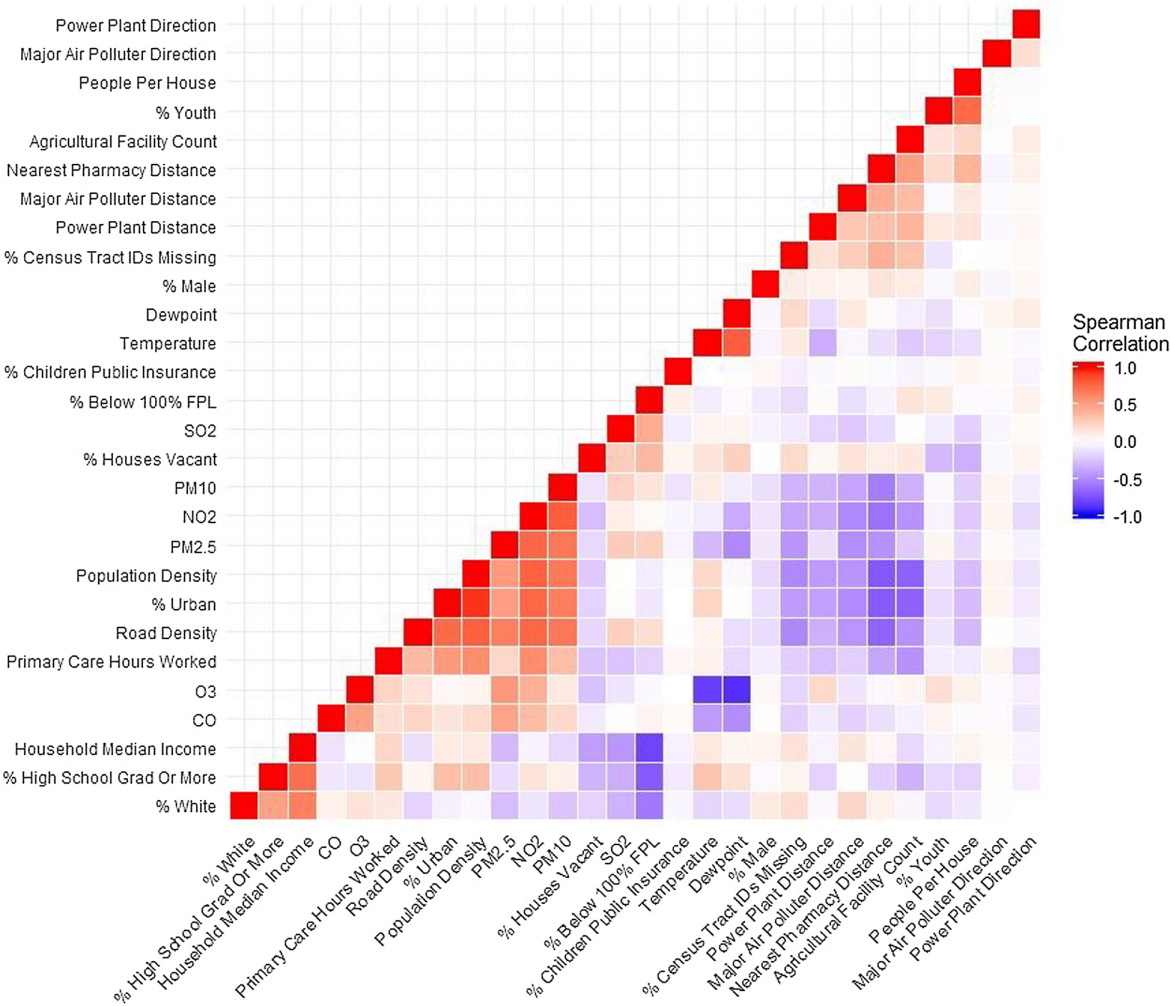
Heatmap showing spearman correlations between variables considered during model building

**Fig. 6:**
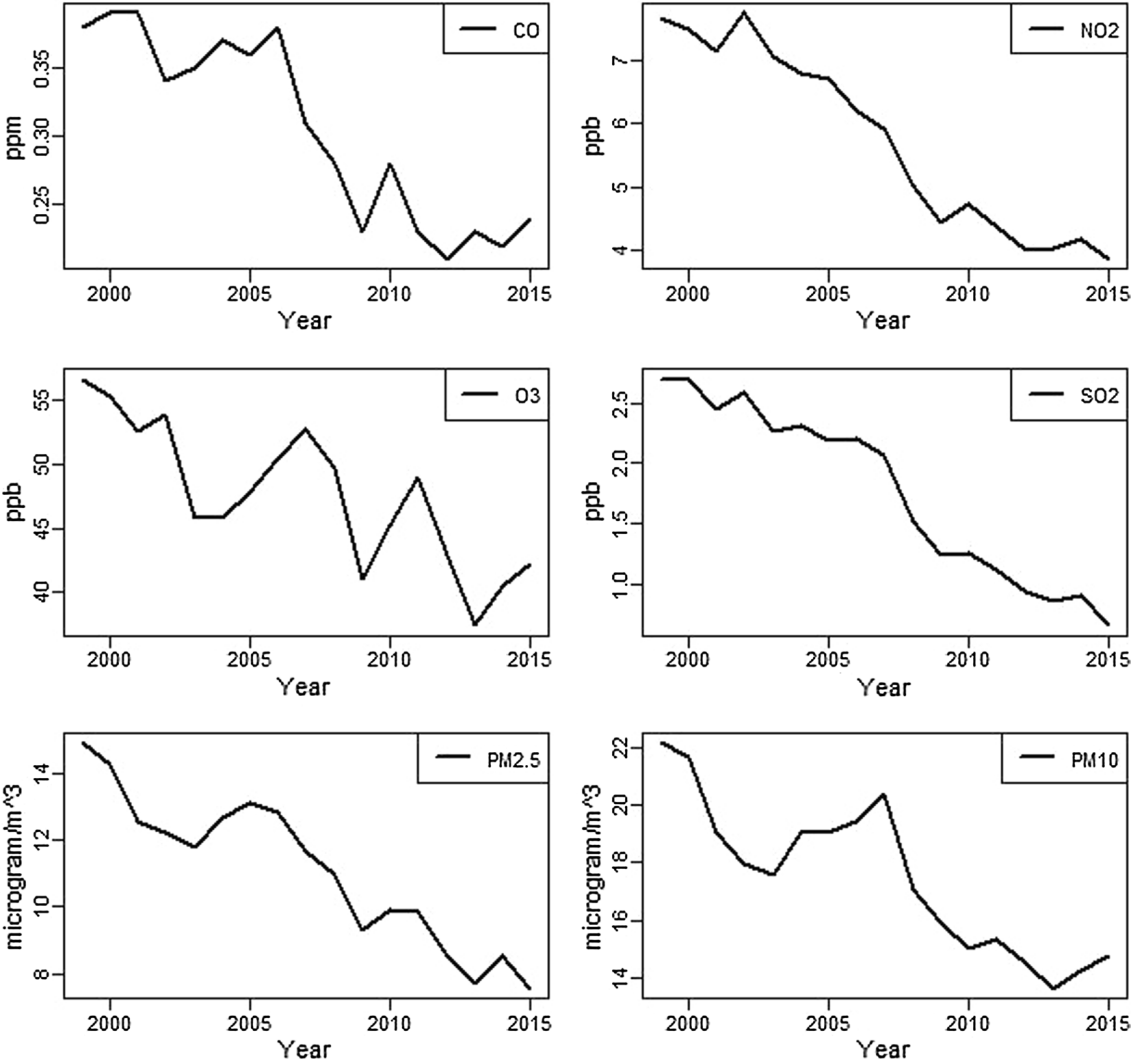
Criteria air pollutant concentrations in South Carolina 1999–2015. Data: Center for Air, Climate, and Energy Solutions (CASES), Environmental Protection Agency (EPA)

A correlation matrix of all census tract socioenvironmental covariates in the form of a heat map was constructed (Fig. 5) to assess relationships between variables. The heatmap visually indicated positive (red) and negative (blue) Spearman correlations for ranked census tracts. In addition, Fig. 6 shows the average annual concentrations for each of the six criteria pollutants included in this study in South Carolina over 1999–2015.

For each model and dataset, respectively, we evaluated the LPML measure of fit (Table 3). Note that Table 3 LPML fit measures can be directly compared only within each dataset (i.e., within-column) and not across Datasets A1 (presumed gold standard) and B. Inclusion of random effects (Model 1a) instead of demographic and weather covariates (Model 1b) was far superior in terms of LPML fit (− 3770.0 to − 4858.5). In addition, including random effects, demographic, and weather covariates in a model (Model 2, LPML: -3756.1) was superior to a model with only random effects (Model 1a, LPML: − 3770.0). By this measure, Model 4 was the best fitting model (LPML: − 3736.7).

**Table 3:** Log pseudo-marginal likelihood (LPML) cross-validated model fit statistics for Models 1–4 for Dataset A1 (includes geographic imputation) and Dataset B (complete cases only)

Model	Description	LPML	
		Dataset A1	Dataset B
1a	Intercept + random effects	− 3770.0	− 3556.1
1b	Intercept + demographics	− 4858.5	− 4603.1
2	Model 1a + model 1b + weather	− 3756.1	− 3542.4
3	Model 2 + variable selection	NA	NA
4	Model 3 + air pollutants + social + environmental + interactions	− 3736.7	− 3543.6

Beta coefficient estimates, standard deviations, and credible intervals were included for Model 4, the final model, for Datasets A1 and B in Table 4. Results from variable selection (Model series 3) conducted on the five datasets (A1–5) that used the areal proportion-based geographic identifier assignment algorithm were consistent. In all five of the datasets, people per house and average annual CO concentration were selected, and in four of the models, distance to nearest pharmacy was selected. Given the consistency of variable selection results, we assumed that Dataset A1 was representative and could be used as the final model.

**Table 4:** Pediatric asthma emergency department (ED) visit relative risk estimates, standard deviations, and credible intervals for a 1-unit increase after controlling for all other factors for the final model (Model 4) for Dataset A1 (includes geographic imputation) and Dataset B (complete cases only)

Description	Dataset A1			Dataset B		
	Coefficient Estimate	Standard Deviation	95% Credible	Coefficient Estimate	Standard Deviation	95% Credible
Model intercept	− 0.307	0.030	− 0.366, − 0.248	−0.560	0.034	− 0.627, − 0.495
Demographics						
Percent of the population white race	− 0.013	0.001	− 0.015, − 0.011	− 0.015	0.001	− 0.017, − 0.014
Percent of the population age 5-19	− 0.063	0.004	− 0.070, − 0.056	− 0.071	0.004	− 0.079, − 0.063
Percent of the population male	− 0.012	0.003	− 0.018, − 0.005	− 0.011	0.004	− 0.018, − 0.003
Percent of the population graduated high school	− 0.013	0.002	− 0.017, − 0.008	− 0.011	0.002	− 0.016, − 0.006
Household median income (scaled by $1 k)	− 0.008	0.001	− 0.011, − 0.006	− 0.011	0.001	− 0.013, − 0.008
Average annual proportion of census tract identifiers missing	0.395	0.084	0.231, 0.561	NA	NA	NA
Weather						
Average annual temperature (°C)	− 0.028	0.010	− 0.048, − 0.008	− 0.032	0.008	− 0.046, − 0.018
Average annual dewpoint temperature (°C)	0.034	0.007	0.021, 0.048	0.016	0.007	0.003, 0.029
Air pollutants						
Average annual CO concentration (ppm)	− 0.148	0.070	− 0.286, − 0.004	NA	NA	NA
Average annual O_3_ concentration (ppb)	0.001	0.001	− 0.002, 0.003	NA	NA	NA
Social						
Distance to nearest pharmacy (km)	0.015	0.004	0.007, 0.023	NA	NA	NA
Average people per house	0.297	0.076	0.149, 0.452	0.521	0.080	0.363, 0.679
Interactions						
CO by O_3_ interaction	0.058	0.010	0.039, 0.077	NA	NA	NA
CO by temperature interaction	0.287	0.090	0.102, 0.452	NA	NA	NA
CO by dewpoint temperature interaction	− 0.261	0.077	− 0.415, − 0.105	NA	NA	NA

In Model 4 for Dataset A1, the demographic and weather measures included as controls were statistically significant. Despite attempting to better characterize neighborhoods by assessing 19 additional socioenvironmental factors of census tracts, the associations between neighborhood asthma risk and race (percent white, *β*: − 0.013; 95% CI: − 0.015,− 0.011), education (percent graduated high school, *β*: − 0.013; 95% CI: − 0.017, − 0.008), and income (median household income, *β*: − 0.008; 95% CI: − 0.011, − 0.006), respectively, could not be explained away. As such, the more white, educated, and wealthy a census tract was, the lower risk for an asthma ED visit it had, controlling for all other factors.

Aside from demographics and weather variables, socioenvironmental variables selected into Model 4 for Dataset A1 included average annual CO concentration (*β*: − 0.148; 95% CI: − 0.286, − 0.004), distance to nearest pharmacy (*β*: 0.015; 95% CI: 0.007, 0.023), average people per house (*β*: 0.297; 95% CI: 0.149, 0.452), and CO interactions with O_3_ (*β*: 0.058; 95% CI: 0.039, 0.077), temperature (*β*: 0.287; 95% CI: 0.102, 0.452), and dewpoint temperature (*β*: − 0.261; 95% CI: − 0.415, − 0.105), controlling for all other factors, respectively. Additionally, though it was not associated with improved model fit, we included a main effect for O_3_ (*β*: 0.001; 95% CI: − 0.002, 0.003) to aid in interpretability because O_3_ significantly interacted with CO. When we removed records with missing census tract identifiers, as in Dataset B, or assigned them based on population proportions, as in Datasets A6-10, socioenvironmental variables selected from Model 3 via variable selection included a very different and smaller collection: only average people per house was significantly associated with asthma ED visit risk. Distance to a pharmacy, CO, and interactions with CO, each of which have strong urban/rural dimensions, were not significantly associated with asthma ED visit risk among children. Furthermore, variable selection for Datasets A6–10, using proportions of populations as assignment probabilities, showed that only average annual CO concentration was selected consistently, and average people per house and nearest pharmacy distance were not selected. The contrasting results seem to be consistent with the patterns of increased geographic missingness of ED visits in rural areas (Table 2, Fig. 3), also reported by others [8].

## Discussion

The examination of relationships between neighborhood factors and health outcomes remains a difficult task for epidemiologists and public health researchers [58]. This study sought to identify an improved methodological approach to common challenges encountered by environmental health researchers, including missing small-area geographic information, multiple correlated neighborhood covariates, and unmeasured confounding factors at multiple spatial scales. We strove to develop a small-area, spatio-temporal methodology that would allow us to disaggregate complex multidimensional data often used in neighborhood studies into a subset of the most predictive components.

### Methodologic goal: geographic information and variable selection

Our primary methodologic goal was to identify differences in final models using variable selection on datasets that employed different methods of handling missing census tract identifiers. In Table 4, the final model for Dataset A1, which *included* geographic imputation via areal proportion probabilities of assignment, was different than the final model for Dataset B, *without* imputation, and different than the final models for Datasets A6–10 that used population proportions as assignment probabilities. Also of interest was the consistency of variable selection results for five independent assignments for missing census tract identifiers using our areal proportion imputation algorithm. We showed that, despite the multiple independent assignments, variable selection identified predictors consistently in the spatio-temporal BHM predictive framework. We believe that our results were consistent across datasets because any “small misses” (an incorrect assignment of a record to a specific census tract within a ZIP code) were compensated for by the smoothed structure of the spatially correlated random effect term, $u_{i}$. As such, while true geocoding accuracy within ZIP codes was relatively unimportant for identifying key predictors in the presence of the smoothed spatial effect, maintaining records via the *areal proportions* imputation approach was important. As the mechanisms causing the inability of addresses to be linked with census tracts were non-standard address types that are more strongly associated with rural areas, our preference was to use areal proportions that assigned higher probabilities to rural census tracts. As such, assignments of missing census tracts based on population proportions likely did not characterize rural risk factors as well, given that that procedure was more likely to assign missing tracts to denser, urban areas. Application of the algorithm was critical for permitting records with missing census tracts to have a valid identifier, thereby retaining them in the analysis. The increased counts produced by this methodology is an advantage, as it offers an enhanced ability (i.e., power) for detecting subtle effects, often critical in studies using ecologic population level socioenvironmental factors.

The estimates for important sociodemographic variables (e.g., race, education, income) identified in other studies were similar between the two final models. However, the model (Model 4) fit to our imputed data (Dataset A1) revealed two additional important predictors: distance to nearest pharmacy and air pollution (CO and its interactions). Without addressing the small-area geographic missingness, we would not have identified these potentially key predictors of asthma ED visit risk. Although additional validation of our methodology is needed across other spatial contexts and datasets, we feel this important finding demonstrates how our approach could be used to enhance and improve precision in similar studies.

Another benefit added by the geographic imputation was the potential for the reduction of bias. This is demonstrated in Table 2, as the records with missing census tract identifiers show higher proportions of African Americans and people visiting rural admitting facilities. Exclusion of such data would generally bias findings toward nonminority groups in urban areas. The handling of missing geographic information was shown to critically influence and change results and the subsequent interpretation of disparity predictors, particularly for rural subpopulations.

### Public health goal: identification of key neighborhood predictors

A public health objective was to improve our understanding of key neighborhood predictors of health risk, specifically asthma ED visits. We found that measures of neighborhood sociodemographic factors and weather conditions were consistently significant in our models (Table 4). Consistent with other contextual health effect studies, census tracts with greater numbers of Caucasians, educated individuals, and higher incomes experienced lower rates of ED visits for pediatric asthma [22, 40, 59, 60].

Additional neighborhood factors associated with increased asthma ED visit risk included increased distance to a pharmacy, increased average number of people per household, and interactions involving CO. “Pharmacy deserts” have attracted scant attention in the context of asthma, though researchers have shown that the ratio of controller-to-total medications predicts ED visits for asthma [61, 62]. Geographic access to specialists and primary care professionals each significantly predicted asthma ED visits in nearby North Carolina, but they were only marginally significant in neighboring Georgia [63]. Our finding of a significant association with people per house may be both a proxy for family living conditions (e.g., crowding) and/or a dimension of neighborhood quality, which is consistent with findings in previous studies [64]. While CO appeared to have an independently protective relationship with asthma ED visit risk, it was found to vary by levels of O_3_, temperature, and dewpoint temperature. Findings of detrimental effects of CO have been predominantly linked with urban areas having high levels above health effect thresholds [65, 66]. However, CO may be complicated by having multiple health effect thresholds, as toxicologic and biologic research has indicated potentially beneficial anti-inflammatory effects of low levels of CO [67, 68]. In addition, SC is generally characterized by having low air pollution levels relative to the United States [69] and other parts of the world, including Europe [70]. Furthermore, our measure of CO was strongly correlated with percent urban, population density, and road density (Fig. 5). Thus, CO may have been also acting as an urban/rural indicator, capturing an effect of urbanized areas having better access to primary and preventive care than rural areas in our study population. In many places in SC air pollution levels may be below *chronic* thresholds for asthma. Shorter temporal scales, such as *days* preceding an ED visit, are more often used in health effects studies [71–74]. Last, we note that calibrating CO exposure models in SC has been problematic due to sparse monitoring in the state, and estimated exposures may therefore have potential for inaccuracies. Regardless, researchers should continue to study the dose–response relationship of ambient CO exposure for both adverse and protective health effects.

Numerous hypothesized or previously identified factors were surprisingly not selected by the variable selection procedure, including distance and direction to nearest power plant and to nearest major air polluting TRI environmental confounders, agricultural facility count, road density, housing vacancy rate, urbanicity, population density, primary care availability, and percent of the pediatric population on public insurance.

## Limitations

There are limitations to this research. The geographic bounds of “neighborhoods” used herein were not community defined. Census tracts, while preferable to much coarser and less population-homogeneous units such as ZIP codes or counties, only served as proxy measures of neighborhoods [6]. Census tracts are proportional to population density, and representing them as points (population weighted centroids) could have been problematic in large and rural census tracts, in particular. This was an ecologic study, and we lacked the ability to easily control for individual covariates and confounding factors due to aggregation for the underlying Poisson count model used. Sub-annual (e.g., hourly, daily, weekly) population mobility was also unaccounted for with these methods using residential billing addresses. Many of the ecologic confounding factors were static, in essence only capturing a mean association over time. We could not control for current tele-pharmacy usage, and it was possible that some rural residents may already have been using such services. Finally, it was difficult to control for the sub-population that used the ED in place of primary care, in addition to “frequent fliers” who used the ED regularly.

## Conclusion

In this study we addressed major methodologic limitations in spatio-temporal analyses of administrative health care data. We presented a predictive framework for consistently identifying important neighborhood factors using a case study of asthma ED visits in South Carolina. We developed an areal proportion-based geographic identifier assignment algorithm that we used in conjunction with a state-of-the-art spatio-temporal BHM. We addressed missing and misaligned geographic identifiers, multiple correlated covariates, unmeasured confounding factors, and other potential sources of bias. In addition to reducing bias, we increased statistical power for improving precision of estimated associations by utilizing coarser spatial information (i.e., ZIP code identifiers) to maintain records. Models were fit in the NIMBLE package in R, a novel application to health utilization data. We disaggregated complex indexes such as SES and urbanicity/rurality to measurable components with potential for intervention and additionally assessed synergistic effects. This novel small-area, spatio-temporal methodology could be applied to other outcomes and locations, making it an important analytic tool for researchers desiring to leverage ecologic data.

## Data Availability

The health outcome data that support the findings of this study are available from the South Carolina Revenue and Fiscal Affairs (SCRFA) office, but restrictions apply to the availability of these data, which were used under license for the current study, and are not publicly available. Outcome data are however available from the authors upon reasonable request and with permission of the South Carolina Revenue and Fiscal Affairs (SCRFA) office. Covariate data used in this study are publicly available from numerous sources listed in the manuscript.

## Abbreviations

CASES: Center for Air, Climate and Energy Solutions
CI: Credible interval
CO: Carbon monoxide
CPO: Conditional predictive ordinate
DIC: Deviance information criterion
ED: Emergency department
EPA: Environmental Protection Agency
GIS: Geographic information system
ICD9: International Classification of Disease 9
LPML: log pseudo marginal likelihood
MCMC: Markov chain Monte Carlo
MSPE: Mean squared predictive error
NO_2_: Nitrogen dioxide
O_3_: Ozone
PM_2.5_: Particulate matter size 2.5 microns or smaller
PM_10_: Particulate matter size 10 microns or smaller
RR: relative risk
SEA-AIR: SocioEnvironmental Associations with Asthma Increased Risk study
SES: Socioeconomic status
SC: South Carolina
SCBOP: South Carolina Board of Pharmacy
SCDHEC: South Carolina Department of Health and Environmental Control
SCDOT: South Carolina Department of Transportation
SCRFA: South Carolina Revenue and Fiscal Affairs office
SO_2_: Sulfur dioxide
US: United States
WAIC: Watanabe-Akaike information criteria

## Footnotes

1 We used the 2010 US Census determination of urban versus rural [78] in our continuous measure of census tract percent urban. However, urban/rural delineations can be determined differently within the US and globally by other methodologies [79].
