# Correction to: A geographic identifier assignment algorithm with Bayesian variable selection to identify neighborhood factors associated with emergency department visit disparities for asthma

**DOI:** 10.1186/s12942-020-00206-4

**Published:** 2020-04-21

**Authors:** Matthew Bozigar, Andrew Lawson, John Pearce, Kathryn King, Erik Svendsen

**Affiliations:** 1 Division of Epidemiology, Department of Public Health Sciences, Medical University of South Carolina, Charleston, SC USA; 2 Division of Biostatistics, Department of Public Health Sciences, Medical University of South Carolina, Charleston, SC USA; 3 Division of Environmental Health, Department of Public Health Sciences, Medical University of South Carolina, Charleston, SC USA; 4 Department of Pediatrics, Medical University of South Carolina, Charleston, SC USA; 5 School-Based Health, Center for Telehealth, Medical University of South Carolina, Charleston, SC USA

## Correction to: Int J Health Geogr (2020) 19:9 10.1186/s12942-020-00203-7

Unfortunately, the original version of the article [1] contained an error. A typo in the main equation (Eq. 1) has been introduced during the production process. The operator “ = ” in Eq. 1 “log(*θ*_*ik*_) = * α *+* u*_*i*_…” was missing.

The equation should read as:

1 $$\begin{aligned} Y_{ik} & \sim Poisson\left( {\mu_{ik} } \right) \\ \mu_{ik} & = E_{i} \theta_{ik} \\ \log \left( {\theta_{ik} } \right) & = \alpha + u_{i} + v_{i} + w_{j} + g_{k} + \varvec{\beta^{\prime}}_{1} \varvec{X}_{ik} + \varvec{\beta^{\prime}}_{2} \varvec{X}_{j} \\ \beta_{*} & \sim Normal\left( {0,\tau_{*}^{ - 1} } \right) \\ \tau_{*}^{ - 1} & \sim Gamma\left( {2,1} \right) \\ \end{aligned}$$

The original article has been corrected.
